# A New Source of Heterogeneity in Comparative and Translational Clinical Trials: The “Border-Time” Bias

**DOI:** 10.3390/cancers14215265

**Published:** 2022-10-26

**Authors:** Mariachiara Santorsola, Michele Caraglia, Guglielmo Nasti, Alessandro Ottaiano

**Affiliations:** 1 Istituto Nazionale Tumori di Napoli, IRCCS “G. Pascale”, Via M. Semmola, 80131 Naples, Italy; 2 Department of Precision Medicine, University of Campania “L. Vanvitelli”, Via L. de Crecchio, 7, 80138 Naples, Italy

**Keywords:** clinical trial methodology, bias, precision medicine, biomarkers, translational trials

## Abstract

**Simple Summary:**

Target-oriented drugs are profoundly changing the anti-cancer treatments. Progression-free survival is a primary or co-primary endpoint in the large part of comparative and translational clinical trials about target-oriented anti-cancer agents. In this context, the time before treatment start and the reassessment of disease (“border-time” bias) are an underestimated source of heterogeneity with unpredictable and uncontrollable impact on final evaluation of progression-free survival.

**Abstract:**

The use of target-oriented drugs is profoundly changing the anti-cancer treatments. This new and expanding therapeutic context relies on the translation of biomarkers expression (laboratory testing) into clinical practice (treatment). Progression-free survival is a primary or co-primary endpoint in the large part of comparative clinical trials about biologic anti-cancer agents. Here, we describe the “border time” bias represented by specific time points and intervals that are an underestimated source of methodologic heterogeneity and can contribute to wrong evaluation of time-to-outcome. These issues are concentrated at the beginning (head: pre-screening and screening activities) and at the end (tail: modalities of disease reassessment) of the anti-cancer treatment and can represent a time-related bias. Reporting, and ideally shortening, the time spent in pre-screening and screening activities with synthetic and innovative methodological tools as well as more harmonized rules about timing of disease reassessment can contribute to reduce, or even prevent, this bias in clinical studies.

## 1. Introduction

The use of target-oriented drugs is profoundly changing the anti-cancer treatments. A new and expanding therapeutic context relies on the translation of biomarkers expression (laboratory testing) into clinical practice (treatment) [1]. However, the confirmation of drug efficacy is ultimately based on performing phase III comparative clinical trials comparing new drugs to standard therapy or placebo [2,3]. 

Several biases can affect the interpretation of the results of these studies. Therefore, a new and independent discipline was born to identify biases and to provide methods to overcome them with the final intent to increase the reliability and generalizability of results: clinical trials methodology [4]. Collaboration between Oncologists and Statisticians is pivotal to prompt evolution of clinical trial methodology. “Stratified randomization” is a milestone of clinical research as it balances, in the different treatment arms, any patient-related prognostic factors known to influence the outcome. Unfortunately, unknown prognostic factors are likely to be “approximately” balanced. In this context, statistical inference and sub-group analysis strongly help to provide good estimates of result reliability and data interpretation [5]. However, caution should be taken in subgroup analyses interpretation mainly because of their limited statistical power and intrinsic risk of positivity for a "multiplicity" effect.

Progression-free survival (PFS) is a primary or co-primary endpoint in the large part of comparative clinical trials about anti-cancer agents including also “new generation” studies with biologic drugs. PFS represents the time elapsed from randomization to the first radiological evidence of tumour progression. In this context, we have identified specific time points and intervals that can contribute to wrong PFS evaluation. These issues are concentrated at the beginning and at the end of the anti-cancer treatment and can represent a time-related bias.

## 2. Time-Related Biases

**Figure 1:**
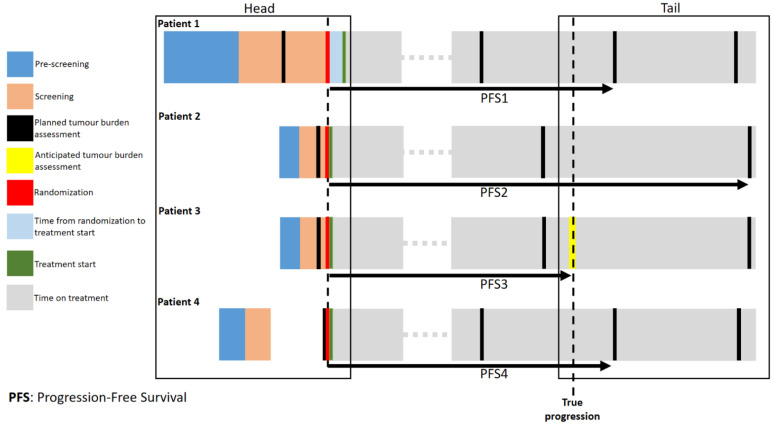
Description of “border time” bias frame-times.

Two moments in clinical trials about target-oriented drugs are affected by heterogeneity with unpredictable and uncontrollable effects on final evaluation of PFS: (i) the moment before treatment starts and (ii) the reassessment of disease (head and tail, respectively, of the “border time” bias, Figure 1). 

### 2.1. Head of “Border Time” Bias

The first is the sum of two frame-times: (1) pre-screening activities including evaluation of general characteristics (age, performance status, medical history and baseline comorbidities, Specialist-driven diagnostic procedures, wash-out from medications, etc.) to determine clinical eligibility, (2) subsequent screening procedures for biomarker analysis (evaluation of mutations, rearrangements, protein expression, etc.) to determine the final molecular eligibility. The duration of both times is stated in protocol documents, and it may vary from one to six-eight weeks. More stringent rules are applied for the time elapsing from the radiological evaluation of disease to treatment start which should generally not exceed 28 days.

Four scenarios are envisaged and described in Figure 1. In Patient 1, the time elapsed from the first pre-screening assessment to treatment start is larger than in patients 2, 3 and 4, allowing a greater chance to tumour progeny to growth and become heterogeneous. Therefore, this is a biological detrimental effect (head of the bias) difficult to quantify and control. In this case, the source of variability can be attributable to pragmatic and context-related reasons (related to hospital logistics and expertise) as well as to specific clinical characteristics (clinical complexity of the patient). In some cases (Patient 4 scenario), the patient has undergone to a resection of the tumour in the past and the clinicians has already the information about the molecular signatures that are inclusion criteria for the study.

### 2.2. Tail of “Border-Time Bias”

The tail of the “border-time” bias relies on modalities of reassessment of the disease. Assuming that the study arms are equivalent and that they have the same true progression (progression at the same time points), different timings of radiological tumour reassessment can produce different PFSs. In fact, Patients 1 and 2 differ only for the timing of instrumental planned restaging, more frequent in patient 1 than 2. In Patient 3, the restaging is anticipated based on a clinically suspected progression at discretion of the Oncologist (a frequent scenario in clinical practice). Different timings of radiological tumour reassessments are a source of inter-trial heterogeneity (PFS1 vs. PFS2). Anticipated reassessment is a source of intra-trial heterogeneity (PFS2 vs. PFS3).

The differences between the PFSs depicted in Figure 1 can exceed 4–8 weeks. Therefore, large multicentre comparative trials reporting small, but statistically significant PFS differences are more prone to the effects of this “border-time” bias, which is not included in the stratified randomization procedure [6,7]. 

## 3. Conclusions

Prevention and/or awareness about biases in clinical trials can ameliorate the interpretation of the results. Here, we describe for the first-time the “border-time” bias. Reporting the time spent in pre-screening and screening activities with synthetic and innovative methodological tools and more harmonized rules about timing of disease reassessment (including the fraction of patients who anticipates reassessment) by treatment arms can be important to give more definitive and statistically correct information about clinical trial. Another possible intervention is the potentiation of speeding the procedures applied for the molecular characterization of the tumours. To this regard, the time spent in screening can be important not only for the influence on the outcome of enrolled patients, but also for the selection bias, which is consequence of the long time spent in screening procedures. This can be relevant especially in settings characterized by a dismal prognosis and a concrete risk of clinical deterioration. Improving the access to technological platforms that give the diagnostic outputs in short time (i.e., next generation sequencing -NGS-, nanostring and others) can become crucial to reduce and even prevent this bias in comparative and translational trials. In fact, one of the most important tools to minimize this bias could be an objective quantification of tumour heterogeneity and tumour burden, which are strictly interrelated. Ideally, this achievement could contribute to minimize this "border-time" bias by improving the clinical and biological homogeneity of patients. While this issue was essentially "science-fiction" until the recent past, it can now be pursued as a result of improved technology (radiomics, NGS, artificial intelligence).

## Data Availability

Not applicable.
